# Building support for children and families affected by stroke (BUILD CARE): Study protocol

**DOI:** 10.1371/journal.pone.0308765

**Published:** 2025-02-05

**Authors:** Maja Kevdzija, Lisa Bartha-Doering, Ruth Heying, Ann Heylighen, Andrea Jelić, Pleuntje Jellema, Anna Franziska Kalhorn, Sophie Mandl, Gesine Marquardt, Birgit Moser, Magdalena Muszynska-Spielauer, Els Ortibus, Anna-Theresa Renner, Anne-Sophie Schoß, Piet Tutenel

**Affiliations:** 1 Department of Building Theory by Design, Institute of Architecture and Design, Faculty of Architecture and Planning, TU Wien, Vienna, Austria; 2 Department of Pediatrics and Adolescent Medicine, Comprehensive Center for Pediatrics, Medical University of Vienna, Vienna, Austria; 3 Pediatric Cardiology Department, Department of Cardiovascular Developmental Biology and University Hospitals of KU Leuven, KU Leuven, Leuven, Belgium; 4 Research[x]Design, Department of Architecture, KU Leuven, Leuven, Belgium; 5 Building Physics and Sustainable Design, Department of Civil Engineering, KU Leuven, Leuven, Belgium; 6 Department of Public Finance and Infrastructure Policy, Institute of Spatial Planning, Faculty of Architecture and Planning, TU Wien, Vienna, Austria; 7 Social and Health Care Buildings and Design, Faculty of Architecture, TU Dresden, Dresden, Germany; 8 Institute of Applied Statistics, Johannes Kepler Universität Linz, Linz, Austria; 9 Pediatric Neurology Department, Department of Development and Regeneration, KU Leuven Child and Youth Institute and; University Hospitals of KU Leuven, KU Leuven, Leuven, Belgium; Chinese Academy of Medical Sciences and Peking Union Medical College, CHINA

## Abstract

Childhood stroke is a rare condition that significantly impacts affected children and their families due to children’s frequently persisting cognitive, physical, and behavioural problems. Existing research on adult stroke shows that the built environment plays a major role in their (partial or possible) recovery and everyday life, but its role has been overlooked in children population. This multidisciplinary research study aims to investigate (1) the role of informal (i.e., home, neighbourhood, school) and formal (i.e., hospital, rehabilitation clinic, outpatient clinic) care environments in the everyday life of children and families confronted with childhood stroke; (2) the families’ financial burden resulting from this rare disease and their economic situation likely affecting the access to care, informal care provision and ability to carry out home modifications.; as well as (3) children’s stroke-related cognitive impairments affecting their experiences of the built environment and their care. The research consists of the preparatory research phase, where existing materials are explored, and three main research phases, each related to one or multiple project objectives. A multi-method approach is adopted, including qualitative (in-depth interviews and participatory creative methods) and quantitative (online questionnaire and cognitive assessments) research methods. Participants are children affected by stroke and their families in Austria, Belgium and Germany. This is a 3-year project that will continue until the end of August 2025. Ethical approvals in all countries were obtained at the time of protocol submission, and data collection for all three research phases started in the second half of 2023 and is currently ongoing. This project will offer first insights into the role of built (care) environments in the experiences of families affected by childhood stroke. Findings are expected to deliver information on their design to improve the life of children affected by this rare disease and their families.

## Introduction

The Building Support for Children and Families Affected by Stroke (BUILD CARE) research project aims to investigate the role of the built environment in the everyday life of children affected by childhood arterial ischemic stroke and their families, which is a rare disease. This project is conducted by researchers from three disciplines (architecture, health economics, and medicine/cognitive neuroscience) in three European countries (Austria, Belgium, and Germany). This study protocol is a result of the collaborative multi-disciplinary and multi-partner effort to develop a shared research framework for different research stages, such as ethics applications in three countries, participant recruitment strategy, the ethical and practical aspects of data collection (including preparatory research), and interdisciplinary synthesis of research results (e.g., in the form of design recommendations and user profiles).

This protocol paper outlines the research aims, methods and procedures developed to investigate this complex research topic. The development of this study protocol started during the research proposal development as a collaboration of the involved multi-disciplinary team. Subsequently, it underwent modifications to conform to the ethical requirements of each participating country, resulting in the version presented here. This protocol version covers most of the planned research approach within the BUILD CARE project. Research plans described under ‘Planned further steps’ are contingent on the results of the research phases presented below, with the methods and procedures being refined as the project progresses.

### Importance of the built environment

The term ‘built environment’ refers to human-made surroundings that provide physical spaces for various human activities. It plays a crucial role in meeting society’s basic needs, such as providing places to live, work, learn, travel, and enjoy leisure. A thoughtfully designed built environment can promote a more equitable society by accommodating the diverse needs of individuals and groups [1]. The built environments’ role during recovery in the adult stroke population is being increasingly recognised: inadequately designed environments of formal care facilities can cause feelings of loneliness [2], loss of control and general inactivity [2–4]. While home environments often need to be modified for home rehabilitation and everyday life after stroke [5–7], neighbourhoods and outdoor environments can present new challenges [8]. At the same time, research with children and young people in other patient populations highlights that they have highly specific characteristics and related needs in the hospital environment [9,10] and may perceive healthcare facilities as their daily environment rather than ‘special environments’ [11]. Given that the built environment plays an important role in the experiences of adults with stroke and also in other young patient populations, exploring the role of the built environment in the everyday life of children and families affected by childhood stroke deserves special attention.

Furthermore, the path towards (partial or possible) recovery is highly individual for each affected child due to the condition’s rarity, complexity, and often delayed diagnosis [12,13]. A Europe-wide model for long-term support for young people who have suffered a stroke is still lacking [14]. Children affected by stroke rarely receive intensive inpatient rehabilitation following hospitalisation, and although tertiary care is usually available when the child is diagnosed, this support diminishes over time [14]. Considering the trend towards outpatient services in countries like Austria, Belgium, and Germany [15], patients and their families are likely to encounter a wide variety of care environments for treatments and therapy while their recovery paths are still greatly unexplored. Moreover, since childhood stroke is a rare disease, healthcare facilities where children receive care are likely not addressing the special needs of this patient population.

Children are assumed to have a higher recovery rate after stroke compared to adults, which is frequently not the case [16]. Even after specialised rehabilitation, stroke-affected children may still need ongoing support in various aspects of self-care, such as washing, dressing and bathing [17]. Parents can feel abandoned upon their return home, not knowing how to care for their child [18,19]. Also “hidden” behavioral changes and learning disorders were frequently reported that often went unrecognized by authorities, resulting in feelings of helplessness and a need for support among parents [20]. The nature and impact of home modifications needed to alleviate disabling circumstances resulting from a stroke have not yet been studied in families’ everyday life. In addition, a considerable economic burden falls on families due to out-of-pocket and indirect expenses [21]. This is especially difficult for families with low incomes and is aggravated by the fact that studies recognised the strong influence of socioeconomic background on the cognitive outcome after childhood stroke [22].

Most stroke-affected children experience persistent cognitive, physical and behavioural problems that can affect their quality of life [23–25]. Typical consequences of a childhood stroke include one-sided weakness (hemiparesis), ataxia, seizures and visuoperceptual deficits [25]. Complex cognitive skills, such as attention, executive function, visuoconstructive skills, processing speed, language, and working memory, have also been found to be affected by stroke [26,27]. Since children – like adults – interact with the built environment through their body and its senses, these interactions (e.g., spatial navigation and orientation) are affected by stroke-related body changes. It is unclear how these relate to their experiences of the built environment.

### Identified research gaps

There are many areas of limited knowledge related to childhood arterial ischemic stroke [25]. The role of the built environment in everyday life in various care settings has been largely overlooked in relation to childhood stroke. The existing studies fail to consider how buildings and their spatial organisation impact the everyday life and well-being of children affected by childhood stroke and their family members. Given the long-term financial pressure on the families, it is furthermore highly relevant to estimate the (lifetime) costs of a childhood arterial ischemic stroke for the affected families in terms of lost income and home modifications, and this knowledge is currently lacking. Previous research also does not examine how motor, cognitive, visuospatial, and other impairments resulting from a stroke affect children’s experiences and interactions with the built environment. This currently lacking knowledge is needed to provide support for families in the future.

Another major gap in existing research is that children’s perspectives and experiences of their family as a unit (including children, possible siblings, and (grand)parents) are rarely considered. Children and adults ((grand)parents, healthcare professionals) participate in care practices together. The importance of considering children’s relations with family members, peers, and other adults is already recognised outside of formal care contexts (e.g., play environments, public spaces) through the shift from designing ‘child-friendly’ to ‘family-friendly’ environments and intergenerational spaces [28]. Therefore, giving voices to children and considering the family as a unit are essential.

### Project objectives

This project, focusing on the family as a unit, aims to explore the everyday life of children affected by stroke and their families from the built environment perspective. This topic has not been previously researched to the best of our knowledge. The role of both informal (i.e., home, neighbourhood, school) and formal (i.e., hospital, rehabilitation clinic, outpatient clinic) care environments in their everyday life are examined.

The BUILD CARE project adopts a multidisciplinary approach and strives for an interdisciplinary synthesis of research results to contribute to the identified complex research gaps and explore multiple factors influencing the experiences of children with stroke and their families. This approach is reflected in the project team structure and the outlined research objectives.

The project team includes architectural researchers with specific technical know-how and research expertise in healthcare environments (TU Wien, KU Leuven, TU Dresden), who have in-depth knowledge about stroke (in children); experience conducting architectural research on (care) environments with user groups considered vulnerable, including stroke patients; expertise in unfolding user perspectives and their experiences in the built environment, including through participatory research with children; and experience in disseminating and incorporating research results (in)to the design of (health)care settings and built environments more broadly. The project team also involves experts in economic evaluations of formal and informal healthcare interventions (TU Wien) and clinical experts in developmental cognitive neuroscience, neurology, and pediatric cardiology (MedUni Wien and University Hospitals of KU Leuven). This protocol addresses the identified research gaps by focusing on the following objectives:

**Objective 1:** To gain a profound understanding of how formal and informal built care environments hinder and support the everyday life of children with stroke and their families;
**Objective 2:** To examine the children’s health-related quality of life and families’ long-term financial burden due to home modifications, out-of-pocket and opportunity costs;
**Objective 3:** To examine children’s post-stroke cognitive impairments affecting their spatial experiences and care.

## Methods

### Study design

This study has a multi-method design with several types of qualitative and quantitative data collected in parallel and analysed separately. Data are collected at five research sites in three countries (TU Wien and MedUni Wien in Austria, KU Leuven and UZ Leuven in Belgium and TU Dresden in Germany). We investigate the family as a unit (including children, adolescents, possible siblings(grand)parents), following their path post-stroke and mapping the landscape of care (formal and informal) they encounter. To better understand the reciprocal relationship between a user and a building, different strategies are employed:

- Qualitative participatory research using ethnographic methods allows gaining nuanced insights into how children and families experience and interact with the built environment and into how they adapt it in use.
- An online questionnaire examines the economic burden on families caused by home modifications and opportunity costs for all studied countries.
- Cognitive assessments further inform the understanding of children’s experiences and interactions with built care environments.

**Fig 1:**
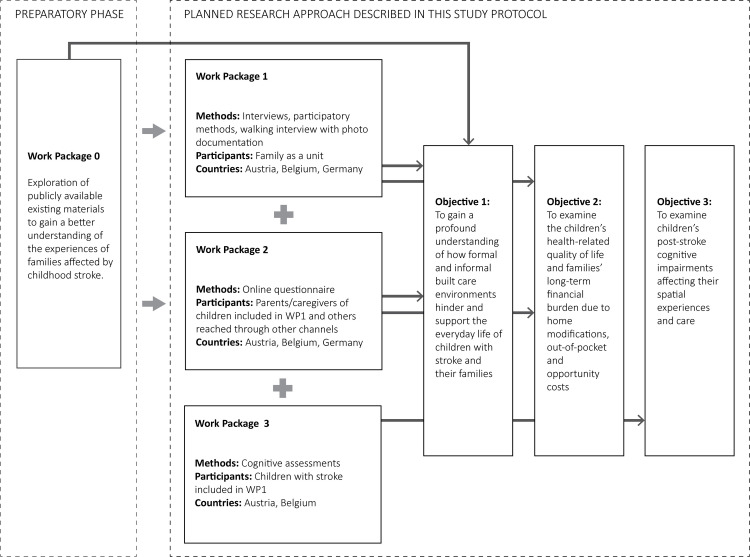
A diagram showing the preparatory research phase (WP0) and three main Work Packages and how they connect to the research objectives.

The research approach consists of Work Package 0 as the preparatory phase that builds the foundation for developing this study protocol and three main research Work Packages (Work Package 1-3) conducted in parallel (Fig 1). Each of the three main research Work Packages relates to one or more research objectives.

### Work package 0 (preparatory research phase)

In this research phase, publicly available existing materials such as autobiographical books/stories, children’s books, journal articles and YouTube videos were explored to gain a better understanding of the experiences of families affected by childhood stroke. As childhood stroke is a rare condition, these sources were invaluable for gaining insights into the families’ everyday experiences and the built (care) environments they encounter. These materials were collected by all involved architectural researchers in a non-systematic way. They included mainly stories of families (from the perspective of parents) depicting how they coped with being confronted with the stroke diagnosis of their child and their recovery journey and experiences. Accounts were included if they were: explicitly about a child with stroke or someone’s own experience of childhood stroke; in English or German; and available to the general public. Both children with childhood stroke and perinatal stroke were included. During this Work Package, architectural researchers in all partner countries adopted different approaches to exploring these materials (e.g., which built environments played a role in the narratives, what emotions were conveyed, and whose voices were heard) [29].

The findings of this preparatory phase supported our approach, especially in refining the planned research topics and interview questions for field research with the families. Valuable information was gained to consider how children with different conditions could participate in this research. The results of the preparatory phase helped us emotionally prepare for the study by developing sensitivity and empathy for this vulnerable participant group [29]. Particularly for the architecture researchers it was valuable to gain insight into the multiple and wide-ranging disabilities children with stroke deal with. Also, the organisational complexity for families combining schooling and care/therapies was important to consider in light of the additional burden of participating in research. This preparatory phase helped us to personally anticipate both the (characteristics of the) people and situations we would encounter in the field. This exploration also led to shaping a specific research focus for Work Package 1 in each partner country, explained in Work Package 1 - Part 3.

### Planned research approach

Building on what we learned in Work Package 0, the three Work Packages that comprise this study protocol are described here, including the planned participants, methods, procedures, and data analysis approach.

### Work package 1

#### Participants.

The inclusion criteria to recruit participants for Work Package 1 are 1) children who have suffered a single arterial ischemic stroke in one hemisphere at least 12 months prior to inclusion; 2) they are between 6 and 14 years old, and 3) children and their participating family members are proficient in German (study sites in Austria and Germany), Dutch, French or English (study site in Belgium). As the participants of this work package also participate in Work Package 3 (cognitive assessments), exclusion criteria used in testing visuo-cognitive abilities are also adopted in this work package. Families with insufficient understanding of the German, English or Dutch language will also be excluded to ensure the validity and usability of the data gathered through the cognitive tests and to minimise possible frustration in the participating children.

The participant recruitment in Austria is facilitated through the Medical University of Vienna, in Belgium through the University Hospitals Leuven and in Germany through a patient advocacy organisation SCHAKI e.V. Additional participants can be recruited through the online questionnaire in Work Package 2 as one of the questions asks the participants to leave their contact information in case they are interested in participating in the interviews. We aim to include 10-15 families of children affected by stroke in each participating country (Austria, Belgium and Germany). The medical professionals involved ensure that participants are included in the study based on the inclusion and exclusion criteria.

#### Methods and procedure.

To explore the role of informal (i.e., home, neighbourhood, school) and formal (i.e., hospital, rehabilitation clinic, outpatient clinic) care environments, the architecture researchers in each country (Austria, Belgium, Germany) will visit participants’ homes, conduct semi-structured interviews, and use methods attuned specifically to involve children. The investigation builds on health geography’s notion of ‘landscapes of care’ [30], which allows taking different scales into account while exploring spatial aspects of social, embodied, and organisational nature linked to (in)formal care and caring relationships. Here, care is defined as bidirectional or reciprocal and includes physical interventions enacted by professionals and all other ((in)formal, emotional/practical(un)paid) situated acts of support, including self-care [30,31]. We consider those affected by stroke - children(grand)parents, siblings(health)care professionals – as user/experts [32] and put their experiences centre stage.

Participatory creative methods are used to explore and (re)present children’s experiences in relation to the built care environment. Participatory visual methods (e.g., photovoice) have a long tradition in community- and development research and have been successfully used in, a.o., education, domestic and (health)care settings with children and youth [9,33–35]. These methods invite participants to express themselves in ways other than verbally (e.g., by making a physical object or collage or sharing an experience) [36–38]. Using a variety of methods enhances the feasibility of responding to unpredictable participants’ circumstances while offering a degree of choice, making it more likely that they will find a suitable format to articulate their experiences.

Work Package 1 has four non-sequential parts (Table 1), which will be carried out during multiple meetings and various research activities with the participating families over a period of 3-6 months. Given that care experiences and practices are shaped by socioeconomic, structural, and temporal processes at different locations and scales [30], these aspects will be included while exploring the landscapes of care. The four research parts are selected to investigate the families’ economic and social circumstances and the spatial, physical and emotional aspects of caring. Each part aims to map various aspects of the families’ multifaceted care landscape. Part 1 looks into their socioeconomic situation, the(ir) child’s well-being, and the locations/institutions visited for formal care. Part 2 aims to better understand the(ir) child’s abilities and disabilities after a stroke, which influences how they experience and interact with the built environment. Parts 3 and 4 delve deeper into the families’ experiences of the informal and formal care environments to identify where, when and how care is provided.

**Table 1:** Work package 1 – participants, parts and methods.

Participants	Method	Parts	Description
Children with stroke and their family members 10-15 families in each country	Interviews	Part 1: Identical core questions in each country	Demographic questions In general, how would your child rate her/his health? Thinking about the last week, has your child felt fit and well? During the last week, how much time did you spend providing practical support to your relative that would not have had to be performed if she/he were in good health or if she/he could have done it independently? Did you reduce your working hours due to your relative’s disease/condition (e.g., to care for him/her)? Which places/settings/institutions have been visited or attended for care after a stroke? In what places does it not matter that your child had a stroke?
	Mappings	Part 2: Identifying child’s strengths and disabilities	Mappings are generated for each child affected by stroke using Part I of the Housing Enabler Tool (www.enabler.nu/download.html, “Functional limitations and dependence on mobility aids”)
	Semi-structured interviews and participatory methods attuned specifically to involve children.	Part 3: Specific focus in each country	Germany: the landscape of care post-acute state of childhood stroke; family’s experiences of journey through healthcare Belgium: The built environment in family’s everyday life Austria: Emotional well-being of children and families
	Building visit; audio-recorded conversation and researcher-made photos and floorplan	Part 4: Documenting experiences and interactions	Accompanying the family to an important building in their everyday life with the main question: “Show me around this place/building”. The following steps are used as a guide for the photos: (a) situations that are impossible to overcome; (b) situations that require assistance; (c) situations that require personal tactics; (d) situations that can be dealt with independently; (e) and situations that are comfortable.

In Part 1, researchers in each country conduct a structured interview with the participating families (Table 1). The mutually agreed upon interview guide consists of questions intended to collect participants’ demographic information as well as information about the child’s health-related quality of life (taken from the KIDSCREEN-10 questionnaire, see Work Package 2), time for providing care/time lost from work (taken from the CIIQ questionnaire, see Work Package 2), and encountered spaces where formal care was provided and their experiences of built environments. These questions were selected to explore various dimensions of the multilayered landscape of care and capture an overview of the situation of participating families in each country, allowing for a comparison of the families’ situations at different research sites. The demographic questions map the educational background and the structure of each family, which might greatly influence their access to care, socioeconomic and gendered inequalities in giving care, as well as the possibilities for implementing home modifications to support the care for the child. Two further questions in Part 1 are related to the family’s economic situation, examining the time spent caring for the child and its impact on the working hours of the family member giving care. Further questions relate to the child’s well-being from the parent’s perspective, aiming to understand each child’s condition, potentially influencing what kind of care they might require. The last two questions explore the spaces that were attended for care after the stroke to identify formal and informal care environments the families encountered and spaces that do not hinder the child because of their post-stroke impairments.

**Fig 2:**
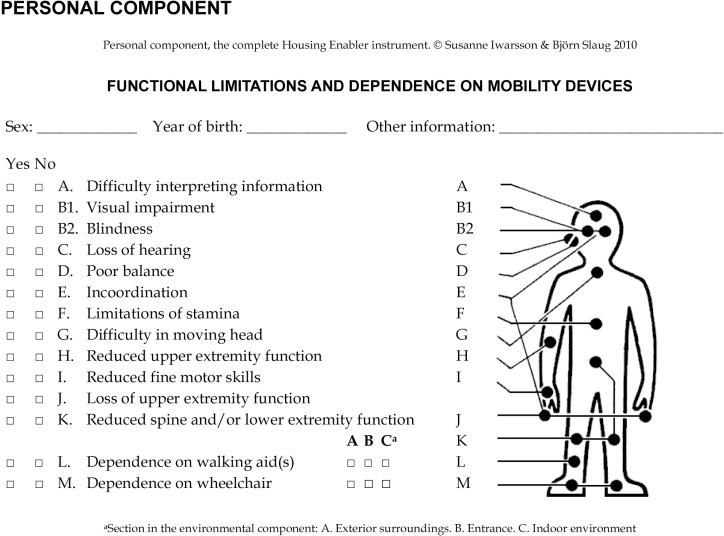
An example illustrating the use of the housing enabler tool to assess functional limitations and dependence on mobility aids [ **39** **].**

Part 2 includes mapping the child’s abilities and disabilities in the conversation with family members to gain a more comprehensive understanding of the post-stroke impairments that might be challenging in their everyday life. To assess the disabilities, we use the first part of the Housing Enabler Tool, an instrument used to evaluate housing accessibility [39], which addresses functional limitations and dependence on mobility aids. The Housing Enabler tool includes a visual representation of a human body with a checklist of various impairments related to different body parts (e.g., severe loss of sight, prevalence of poor balance, incoordination, difficulty in reaching with arms, reliance on walking aids, etc.) (Fig 2) [39]. Where necessary, additional impairments and each child’s perceived strengths and abilities are added by the researchers.

In Part 3, each country adopts a specific, complementary focus for the interviews and participatory methods. This focus is chosen based on the exploration of existing materials in Work Package 0, where the researchers identified topics of importance in the everyday life of children with stroke and their families, as well as on the research interests of the researchers engaged in the study. The focus of researchers in Germany will be on mapping the landscape of care in the post-acute state of childhood stroke and exploring the family’s experiences of their journey through healthcare; the focus in Belgium will be on the built environment in a family’s everyday life; and researchers in Austria will focus on the emotional well-being of children and families affected by stroke.

The transition from hospital discharge to home is mentioned as pivotal in existing materials. In Part 3, researchers in Germany focus on this transition from acute to chronic care at home, known as the post-acute phase. Central to semi-structured interviews is the question of which places, environments, and institutions were sought out for post-stroke treatment. Utilising the participatory method of “photovoice”, children are tasked to photographically document elements impacting various aspects of their everyday life and surroundings. These photographs will serve as a starting point for dialogue between the child and researchers, providing a nuanced exploration of their environment. Children will also guide German researchers through their homes, facilitating a participatory and creative exploration of their built environment. Important places and elements discussed with children and families are marked on floor plans.

In order to gain insight into the role of the built environment in a family’s everyday life, the researchers in Belgium conduct semi-structured interviews with interested family members (individually/ together), asking questions such as ‘How can you tell from this [place/building] that someone who has had a stroke lives or spends a lot of time here?’ And ‘Where do you feel the most ‘out of place’? Why is that, and how do you deal with it?’ Creative methods (drawing, photography) are used to support the interviews; materials will be copied/photographed. In addition, they expand on Part 4 with the aim to include the home and an additional building significant to the child/family. The guiding question throughout these visits is: ‘Can you guide me through this space/building?’. During the walk-along, the conversation is audio-recorded; the researcher and/or participant makes photographs.

Within existing materials, various factors have been identified that can negatively impact the emotional well-being of families. During a home visit, researchers in Austria employ various exploratory methods to understand the families’ emotional experiences in different environments. A key component of the visit involves playing a self-developed participatory, art-based conversation game with the children to explore their spatial-emotional landscape. Additionally, semi-structured interviews are conducted with family members, primarily parents, including general questions about stroke-related symptoms, the impact of potential limitations on a child’s daily life, and the family’s experiences with care environments. Furthermore, a guided tour of the family’s home is conducted with the child to identify potentially hindering or supportive aspects of the built environment.

During home visits, a building important in each family’s everyday life will be selected for Part 4. The researcher(s) will accompany each participating family to this building and document their experiences and interactions with the built environment using a gradient in obstacles, based on Vermeersch & Heylighen [40].

#### Data analysis.

In Work Package 1, multiple data types are collected (e.g., interview transcripts, drawings, photographs), and the researchers in three countries adopt and develop different approaches to data analysis.

In Germany, data, including field notes and floor plan maps gathered during the building visit, are carefully (re-)read and reviewed to ensure a thorough understanding of the visit’s contents. Key themes are marked on the analysis sheet to capture the essence of each interview. Findings are categorised by clustering into themes, with relevant interview fragments linked to each category.

Data analysis in Belgium roughly follows the various stages of analysis outlined by QUAGOL, in order to reconstruct the stories of the participating families in response to the research questions, grounded in the data (for more details see [41]). It is a method to guide qualitative data analysis originally based on a grounded theory approach – two researchers independently identify themes or select scenes in the various data that demonstrate features and qualities of families’ landscapes of care; based on re-viewing observation notes, photos, outcomes of participatory methods and re-reading interview transcriptions. Themes and selected scenes are then iteratively refined. The broader research team discusses each part of the analysis until a consensus is reached.

In Austria, various collected materials, such as interview transcripts, drawings, floor plans and photos, are reviewed and analysed thematically, combining deductive and inductive coding approaches that follow predefined coding guidelines [42]. The focus is on gaining insights into the spatial and emotional landscapes within different environments.

Interview transcripts and other collected data are coded using qualitative software (NVivo), and fieldnotes (gathered through different research activities) are (re-)read to gain a holistic understanding of participants’ experiences.

### Work package 2

#### Participants.

Parents/caregivers of the 10-15 affected children in each participating country included in Work Package 1 are invited to participate in the online questionnaire. Additional parents/caregivers are reached through the project website (https://www.buildcare-project.eu/), a flyer with a QR code leading to the questionnaire, and the patient advocacy organisation SCHAKI e. V.’s newsletter and social media. Because of multiple distribution channels, the questionnaire is expected to reach a broader sample of families beyond the ones participating in WP1. If the participants do not fit the inclusion criteria from WP1, this group can be identified based on the demographic questions in the questionnaire, which will be acknowledged during data analysis.

#### Methods and procedure.

This Work Package includes an online questionnaire focused on the household economics of families with children affected by stroke. This questionnaire was developed to examine the family-incurred costs related to health and long-term care, the opportunity costs of time, and forgone income from labour in three participating countries. Two standardised questionnaires are used: KIDSCREEN-10 [43] and CIIQ [44], with the addition of demographic questions and questions developed by the researchers about the home modifications and (special) equipment needed for informal care (Table 2). The questionnaire is available in German (Austria, Germany) and Dutch (Belgium).

**Table 2:** Work package 2 – online questionnaire.

Participants	Method	Parts	Description
Parents/caregivers of children included in Work Package 1 and additional families reached through distribution channels in all three participating countries	Online questionnaire	Part 1: Demographic questions	Questions related to the age and sex of the child, year of stroke, symptoms of stroke and information about the family(S1 Appendix)
		Part 2: Health Questionnaire for Children and Young People	KIDSCREEN-10 10-item questionnaire measuring general health-related quality of life (HRQoL), parent version
		Part 3: Caregiver Indirect and Informal Care Cost Assessment	Caregiver Indirect and Informal Care Cost Assessment Questionnaire (CIIQ) 13 questions concerning caregiver work status and the provision of paid and unpaid informal care
		Part 4: Questions related to the built environment	Questions related to modifications parents had to make to their homes because of the stroke-related impairments of their child(S2 Appendix)

#### Data analysis.

The responses to the online questionnaire will be analysed using standard statistical methods, including descriptive measures of distribution, central tendency and variability for different groups, and correlation analysis or mean comparison (e.g., t-tests). Nonparametric methods for hypothesis testing, such as the Wilcoxon rank-sum test, will be applied if the normality assumption is violated. Importantly, the results from the KIDSCREEN-10 questionnaire (Part 2) and the CIIQ (Part 3) will be compared to results from other studies to elicit any significant differences between the study population and any other population groups. Finally, the questions regarding necessary home modifications will be analysed qualitatively to highlight the burden of direct non-healthcare costs for affected families.

### Work package 3

#### Participants.

Participants in cognitive tests are the 10-15 children from families included in Work Package 1 in each country. Therefore, the inclusion and exclusion criteria are identical to Work Package 1.

#### Methods and procedure.

The cognitive assessment evaluates abilities and possible deficits in various visuoperceptual tasks (Table 3). The examination of Austrian participants takes place at the Neuropediatric Outpatient Unit of the Medical University of Vienna; participants in Belgium will be assessed at the University Hospitals Leuven. The duration of the cognitive testing varies between 100 and 150 minutes depending on the child’s age, motivation, and attention span. One or more breaks are possible according to the child’s needs. After completing the test, the children receive a certificate for participation.

**Table 3:** Work package 3 – cognitive assessments.

Participants	Method	Parts (Tests)	Description (Cognitive domain)
Children included in Work Package 1	Cognitive assessments	ROCF	visuoperception, visuoconstruction, visual memory
		HAWIK-IV subtests: picture completion, digit span, letter-number sequences	perceptual logical thinking, working memory, analysis of visual information
		Token Test for Children	language comprehension
		FEW-3 subtests: eye-hand coordination, drawing, figure reasoning, shape closure, shape constancy	visuomotor integration, visual perception
		TAP subtests: alertness, shared attention	attention

Note. ROCF, Rey Osterrieth Figure; HAWIK-IV, Hamburg-Wechsler Intelligence Test for Children; FEW-3, Frostigs Entwicklungstest der visuellen Wahrnehmung (English: DTVP-3: Developmental Test of Visual Perception)

#### Data analysis.

Raw scores of cognitive tests are converted into z-scores adjusted for age according to the standardised norms of each test. Cognitive test results will be analyzed alongside the categorical findings from the semi-structured interviews (Work Package 1) using subgroup analyses. Cognitive test results will further be correlated with quantitative results of the online questionnaire on the family’s economic burden (Work Package 2) to inform about possible associations between cognitive (dis)abilities of the child, its family’s economic burden, and the challenges in their built environment. If behavioural data are not normally distributed, nonparametric testing is conducted. The strength of the relationship between continuous variables is examined using Pearson’s correlation coefficient *r* or Spearman’s rank correlation coefficient *r*_*s*_. The significance of correlations is set based on a Bonferroni correction factor with α = .05/number of comparisons.

## Planned further steps

Further research is planned within this project to expand our understanding of the perspectives of healthcare professionals who care for children with stroke as well as to develop and evaluate design recommendations and user profiles that may be used to support the design of built environments that families encounter.

### Formal care facilities as workplaces

Based on the previous Work Packages, the formal care environments and relevant (health)care professionals that are important to the experiences of the children and families will be identified. In-depth semi-structured go-along interviews [45] are planned with (health)care professionals as user/experts [32] and ‘everyday designers’ [46] of their working environments to explore how healthcare facilities are experienced as workplaces and how they provide care for children with stroke. This investigation will examine aspects such as spatial barriers to and facilitators of children’s and families’ well-being and recovery, spatial configuration, distances between spaces, locations and design of common areas (when applicable), specific design elements planned for children, etc.

### Creating user profiles (personas and scenarios)

Based on the research results of previous Work Packages, design recommendations on how to (re)design home and (health)care environments to accommodate spatial requirements specific to childhood stroke will be developed together with a persona set and scenarios that can be used to support design processes. Personas are user profiles [47] developed based on the analysis, interpretation and comparison of quantitative and qualitative empirical data and presented visually and textually [48]. These personas may then be placed into various scenarios, providing detailed settings and situations as a basis for communication between users and designers [49]. Presenting design recommendations alongside personas and scenarios can offer valuable insights to design practitioners and healthcare professionals into different users’ perspectives through vivid descriptions [50].

We will conduct focus group interviews with design practitioners to validate the applicability of developed design recommendations in architectural practice. It is planned that design practitioners representing architecture firms in each of the three participating countries will be invited to participate in focus group interviews. A mix of smaller and larger firms will be sought, as firm size impacts the organisation of the design process. The developed personas, scenarios and design recommendations will be revised based on the results of the focus group interviews.

## Patient and public involvement

While developing the project proposal, the project team had several discussions with representatives of patient advocacy organisations (PAOs), who provided significant input, specifically in formulating research questions and choosing research methods to be used with children with stroke and their families. Their suggestions informed our research approach, selection of different participants and patient inclusion criteria. PAO Rarity United (in Belgium, does not exist anymore) was involved in the proposal development, while these organisations remained involved in the project after the proposal phase: German Stroke Foundation (Deutsche Schlaganfall-Hilfe) as a collaborator and SCHAKI e.V. (Germany’s largest self-help group for stroke-affected children led by a family with a child with stroke) as an active partner.

PAOs are also involved in the project’s advisory board to review the data interpretation, provide patients’ perspectives on the project’s progress, and advise on ethical issues they see arising and consider important throughout the project. (Health)care professionals and design practitioners in each country are also invited to be members of the advisory board, which will follow and evaluate the project’s progress and give new insights and advice to the consortium.

## Ethical considerations

This project involves a vulnerable group (children with stroke and their families) and therefore entails more than minimal risk according to The Economic and Social Research Council (ESRC) Framework for Research Ethics. The research is conducted in accordance with the Declaration of Helsinki [51] and the local guidelines and regulations of each study location. In Work Package 1, children receive an age-appropriate information sheet describing the research study aims and procedure, risks and benefits of participation, personal data protection and the right to withdraw from the study at any time (in Austria for age ranges 6-8, 9-11, and 12-14, in Belgium and Germany for age range 6-14). Their legal representatives receive a detailed information sheet describing the research aims, methods, and implications, the nature of the participation, and any benefits, risks, or discomfort. Sheets explicitly state that participation is voluntary and that the child has the right to refuse to participate and withdraw participation at any time without any consequences. They state how data will be collected, protected, and reused subsequently and describe the procedures implemented in the event of unexpected findings. Children’s assent is obtained, and the legal representatives will be asked for written informed consent before the child’s inclusion in the research (for participating and data processing). We refer to children’s assent to align with the European Commission guidelines [52] but, given the participatory intent of our research approach, value this as equal to their *consent* [53]. In addition, a logbook is used with participants in Belgium to ensure that the researchers remind the child participant of the consent agreements at the start of each research activity. We will monitor children and rely on the participating family members to recognise verbal or non-verbal clues that they may wish to stop participating.

The online questionnaire (Work Package 2) is hosted on the LimeSurvey survey platform, recommended by the Ethical Committee at the coordinating institution (TU Wien) as an open-source tool allowing anonymous data processing. Participants are informed about the research project and its aims, what kind of data will be collected, how these will be processed, and their rights on the questionnaire welcome page before they are asked for consent by ticking the provided box. No sensitive personal data are collected from the participants if they only participate in the online questionnaire. If they also participate in the study at the MedUni Wien, the TU Dresden, University Hospitals/KU Leuven or the TU Wien, additional data regarding the voluntary entry of the study ID issued to each participant will be collected, meaning the questionnaire participant’s identity will be known to the researchers. The participants need to consent to connecting their data from different Work Packages. They can withdraw their consent (to data processing and/or connecting their data) at any time during the data collection by contacting the assigned contact person, and their data will be deleted immediately. Participants can also request information about the processed data at any time.

For cognitive assessments (Work Package 3), after a thorough explanation of the aim and process of the study, assent/consent and information forms are signed by the child and by the authorised accompanying person.

Participant risk is assessed continuously to correspond with changes in methodology and circumstances in the research settings. The interviews (combined with other participatory methods), the questionnaire and the cognitive tests have no significant physical, social, reputational, or economic side effects for children and their families. If an individual does not want to discuss a particular topic, this will be respected. The online questionnaire is also designed so that none of the questions are obligatory to answer and can be skipped by the participants.

The consortium confirms full compliance with national and EU law on the protection of individuals concerning the processing of personal data and that the ethical standards and guidelines of Horizon 2020 will be applied. The research was approved by the Ethical Committees of MedUni Wien (approval number: 2263/2021) in Austria, KU Leuven (SMEC Ref. G-2021-4469) and UZ Leuven (approval number: S67320) in Belgium, and TU Dresden (SR-EK-355082022) in Germany.

## Results

BUILD CARE is a 3-year project which started in September 2022 and is planned to continue until August 2025. Final ethical approvals were obtained in October 2022 in Germany, March 2023 in Austria, and June 2023 in Belgium. Data collection for Work Package 1 started on July 24th 2023 in Germany, August 18th 2023 in Austria, and October 13th 2023 in Belgium. The online questionnaire (Work Package 2) was launched simultaneously in all three countries on May 8th 2023. In Work Package 3, the cognitive assessments started on June 17th 2023 in Austria and are planned to start in the second half of 2024 in Belgium.

## Discussion

### Reflections on the protocol development

Developing the protocol demanded of us - as collaborating researchers - to ‘get on the same page early-on’ through different initiatives, i.e., a partner with relevant expertise educated the others through a seminar format, to, e.g., explore space with children; introduce work done with personas; see examples of the tests used to assess visuospatial perception etc. The timelines of the granted project proposal and the required ethics approvals (with financial repercussions) did not fully align with the time and space required to create an environment where different (sub-)disciplines respect and understand each other’s contributions and work towards common goals.

For example, differences between disciplines (e.g., neuroscience and architecture) are easier to understand than differences within a single discipline. While crossing disciplines, we have to rely on each other’s expertise and approach unfamiliar knowledge and ways of working with a certain openness. With eight architectural researchers from three different institutes, however, it takes time to become aware of and overcome one’s own assumptions and viewpoints about what constitutes ‘good’ architectural research. This process of developing a study protocol helps to see these differences as opportunities to better understand the realities of families affected by childhood stroke. The process of collaboratively writing this protocol paper/article is also an exercise in finding common ground between different disciplines (and different approaches within disciplines), raising new questions about protocol purposes and practices. The resulting study protocol and paper/article, covering part of the research, allows us to build on results and keep learning from each other’s approaches when refining the next steps in research, focussing on follow-up protocols (e.g., to explore care professionals’ perspectives; develop and evaluate user profiles). To ensure validity during this ongoing development we follow Angen’s [54] distinction between ethical and substantive validation. ‘Ethical validation’ – whether the research informs or has the potential to transform practices – is guaranteed by integrating situations in the research set-up that help verify the practical and theoretical relevance of routes taken. We do so by, e.g., presenting intermediate results and possible interventions: to our respective research groups; within the consortium; and, to an advisory board of people with relevant expertise. ‘Substantive validation’ – the ways that the research shows and accounts for prior research, theory and self-reflexivity – is strived for by presenting the research within the boundaries of the different disciplines that inform the research: presenting intermediate results at conferences, but also to the general public,…. Together, both forms of validation help to ensure next steps in the research are always well-considered.

To accommodate researchers at different career stages (PhD, postdoc, assistant professors), it is important that the protocol allows for a comprehensive exploration of the subject matter while leaving room to develop and articulate individual research interests. This is reflected in distinguishing between core and other (interview) questions and each country having its own focus (post-acute care, the built environment’s role in everyday lives, emotional well-being) and approach to data analysis (leaning either towards a more positivist or a more interpretivist approach to qualitative research). Starting from existing materials such as biographies or YouTube videos is also a way to develop sensitivity to the research context while creating opportunities that allow differences between researchers with diverse levels of expertise.

International collaborations implicated that materials needed to be developed in multiple languages (e.g., brochure, ICFs, questionnaire). We decided to create the questionnaire in German and Dutch; however, English and French might have been important additions. We are well aware now that researchers should try to avoid excluding families based on language. In addition, the two selected languages do not allow raw data exchange within the consortium due to limited language skills. Also, decisions regarding dissemination to a broad public will demand multiple (sufficiently nuanced) translations to ensure the accessibility of results and recommendations.

### Expected results and benefits

This project will offer insights into the daily experiences of children with stroke and their families, as well as the role of the architecture of (health)care environments in their health and well-being. It will also further gain scientific knowledge on families’ economic burden resulting from this condition and the post-stroke impairments that the affected children experience. Leveraging families’ experiences will contribute to a nuanced understanding of how spaces affect people’s well-being and help them, (health)care organisations and design practitioners to make care environments more supportive, which is expected to have an important societal and economic impact.

Gaining insights into families’ experiences and developing design recommendations for (health)care environments based on specific needs will contribute to creating more inclusive built environments that promote equitable public health and support children’s voices in architectural design. Results on home modifications and recommendations regarding adaptations will directly benefit people affected by the disease without necessitating actions by a proxy; the families of children with stroke will be able to take action on modifications to their home environment themselves. Translating the gained insights into general design recommendations will help design practitioners and care professionals improve the design quality and use of buildings that accommodate the (health) care of children affected by stroke. Ultimately, we seek to contribute to environments that foster rather than hamper users’ well-being.

Furthermore, the transnational and multidisciplinary collaboration within the project consortium will increase the visibility of our results and ensure that the wider audience (families, PAOs, design practitioners, healthcare professionals, researchers, and educators) can access and use them to improve the everyday life of stroke-affected families. Participatory methods and close collaborations with PAOs ensure the accessibility and broad applicability of project results, which may be extended further through the families themselves.

### Strengths and limitations

There are several strengths and limitations to the presented study protocol. A major strength of the data collection in three countries is the relatively large number of participants (for a rare disease) and the continuous inclusion of PAOs throughout the proposal development and the whole duration of the project. The involvement of three disciplines (architecture, health economics and medicine/cognitive neuroscience) offers a unique view of the investigated topic from different perspectives. Another major strength of the project is the inclusion of the perspectives and experiences of the children themselves (children as user/experts) and the family as a unit. On the other hand, including the family members can also be seen as a challenge, as the experiences of all family members might not be adequately represented.

Another limitation is the significant variation in the post-stroke impairments of the participants in children in different countries (e.g., more acute in Austria and more chronic in Germany), which is reflected in the severity of their impairments and whether they use a specific mobility aid or only have minor visual impairments or cognitive issues. Including such a heterogeneous participant group can also be seen as a strength of the project as children with a greater variety of post-stroke impairments are included, which should offer a better understanding of their experiences with the built environment. In addition to the large variety of impairments, some of the participating children have other and/or underlying health conditions, which brings into question the original aim of focusing purely on childhood stroke and its effect on children’s experiences. Moreover, cognitive testing is only performed with Austrian and Belgian study participants. While there is some secondary information on possible cognitive deficits in the German participants from parental reports, the lack of a standardised neuropsychological examination in this group of study participants is a further limitation of this project. The results on the associations between cognitive data, economic burden, and built environment may not simply be generalized to the German context, as the relationships may differ in Germany.

Furthermore, due to the research approach and the participant recruitment strategy, the participant group is likely skewed towards families of higher socioeconomic status, whereby certain families will be underrepresented. This is true not only for the face-to-face interviews but also for the online questionnaire. To avoid the underrepresentation of disadvantaged families, the analysis of the financial burden of families will be stratified by the parents’ socioeconomic background and weighted if necessary. Despite possible selection effects, an advantage of the online questionnaire is that it is accessible for families independent of their geographical location, and, due to its compact format, it is feasible to fill in despite strong time constraints. A major strength of the online questionnaire is that it incorporates two questionnaires (KIDSCREEN and CIIQ) that have been validated and are frequently used in scientific studies. The latter will enable a comparison between our study population and families affected by other childhood disabilities.

For architecture, we are aware of the bias mentioned above. Still, by focusing on professional and everyday design, we aim to consider the diversity in families’ socioeconomic status (e.g., in formulating recommendations). Also, specifically in developing and decision-making regarding a persona set, we will include the worst-off based on the analysis of household economic data and outcomes of neurocognitive assessments.

## Conclusion

This project will map the landscape of care that children affected by stroke and their families encounter during rehabilitation and (possible) recovery and offer first insights into the role of these environments in their everyday life. Adopting a multidisciplinary approach allows for the comprehensive exploration of various factors affecting their experiences with the built environment. The involvement of participants in three countries enables the inclusion of a larger number of participants affected by this rare disease and offers an international perspective on the researched topic. Findings related to home modifications will directly benefit the affected families, empowering them to take action on modifications to their home environments themselves. The project’s results are expected to inform the accommodation, design, and delivery of care and contribute to improving the everyday life of children and families affected by childhood stroke. Furthermore, they are expected to shed light on the importance of studying the role of the built environment in the lives and experiences of people affected by a rare disease.

## Supporting information

S1 Appendix Work Package 2 – Part 1: Demographic Questions (PDF)

S2 Appendix Work Package 2 – Part 4: Questions related to the built environment (PDF)

## References

[1] Seyedrezaei M, Becerik-Gerber B, Awada M, Contreras S, Boeing G. Equity in the built environment: a systematic review. Build Environ. 2023;245:110827.

[2] Anåker A, von Koch L, Heylighen A, Elf M. “It’s lonely”: patients’ experiences of the physical environment at a newly built stroke unit. Heal Environ Res Des J. 2019;12(3):141–52. doi: 10.1177/1937586718806696 30336696 PMC6637812

[3] Kevdzija M, Marquardt G. Stroke patients’ nonscheduled activity during inpatient rehabilitation and its relationship with the architectural layout: a multicenter shadowing study. Top Stroke Rehabil. 2022;29(1):9–15. doi: 10.1080/10749357.2020.1871281 33423616

[4] Kevdzija M, Bozovic-Stamenovic R, Marquardt G. Stroke patients’ free-time activities and spatial preferences during inpatient recovery in rehabilitation centers. Heal Environ Res Des J. 2022:1–18. 10.1177/19375867221113054 PMC9523820 35850529

[5] Karakasi M, Warren N, Manderson L. Orchestrating home: Experiences with spousal stroke care. Med Anthropol Theory. 2017;4(1):SE–Articles.

[6] Marcheschi E, Von Koch L, Pessah-Rasmussen H, Elf M. Home setting after stroke, facilitators and barriers: a systematic literature review. Health Soc Care Community. 2018;26(4):e451–9. doi: 10.1111/hsc.12518 29210130

[7] Kylén M, Ytterberg C, von Koch L, Elf M. How is the environment integrated into post-stroke rehabilitation? A qualitative study among community-dwelling persons with stroke who receive home rehabilitation in Sweden. Health Soc Care Community. 2022;30(5):1933–43. doi: 10.1111/hsc.13572 34541725

[8] Twardzik E, Colabianchi N, Duncan L, Lisabeth LD, Brown SH, Clarke PJ. Well in in this neighborhood I have walked, not at all: Stroke survivors lived experience in the outdoor environment. Soc Sci Med. 2022;305:1–22. 10.1016/j.socscimed.2022.115107 PMC9310555 35690031

[9] Lambert V, Coad J, Hicks P, Glacken M. Young children’s perspectives of ideal physical design features for hospital-built environments. 2014. 10.1177/1367493512473852 23423998

[10] Peeters K, Jellema P, Annemans M, Heylighen A. How do adolescents affected by cancer experience a hospital environment? J Adolesc Young Adult Oncol. 2018;7(4):488–92. doi: 10.1089/jayao.2017.0116 29583076

[11] Tutenel P, Ramaekers S, Heylighen A, Leuven K. The pavement and the hospital bed: care environments as part of everyday life. J Inter Des. 2022;47(4):3–10. doi: 10.1111/joid.12225

[12] Martin C, von Elm E, El-Koussy M, Boltshauser E, Steinlin M, Swiss Neuropediatric Stroke Registry study group. Delayed diagnosis of acute ischemic stroke in children - a registry-based study in Switzerland. Swiss Med Wkly. 2011;141 w13281. doi: 10.4414/smw.2011.13281 22012483

[13] McGlennan C, Ganesan V. Delays in investigation and management of acute arterial ischaemic stroke in children. Dev Med Child Neurol. 2008;50(7):537–40. doi: 10.1111/j.1469-8749.2008.03012.x 18611205

[14] Norrving B, Barrick J, Davalos A, Dichgans M, Cordonnier C, Guekht A. Action plan for stroke in Europe 2018 – 2030. 2018. 10.1177/2396987318808719 PMC6571507 31236480

[15] European Comission. Country Health Profiles. Public Health. [Internet]. 2023. Available from: https://ec.europa.eu/info/sites/default/files/6._h2020_ethicssoc-science-humanities_en.pdf

[16] Goeggel-Simonetti B, Cavelti A, Arnold M, Bigi S, Regényi M, Mattle HP. Long-term outcome after arterial ischemic stroke in children and young adults. Neurology. 2015;84(19):1941–7. 25862797 10.1212/WNL.0000000000001555

[17] Wales L, Dunford C, Davis K. Following severe childhood stroke, specialised residential rehabilitation improves self-care independence but there are ongoing needs at discharge. Br J Occup Ther. 2020;83(8):530–7. doi: 10.1177/0308022619894870

[18] Grover KS. The self-directed learning experience of mothers whose child has had a paediatric stroke. Int J Lifelong Educ. 2014;33(4):488–503. doi: 10.1080/02601370.2013.876558

[19] McKevitt C, Topor M, Panton A, Mallick AA, Ganesan V, Wraige E, et al. Seeking normality: parents’ experiences of childhood stroke. Child Care Health Dev. 2019;45(1):89–95. doi: 10.1111/cch.12622 30255632

[20] Soufi S, Chabrier S, Bertoletti L, Laporte S, Darteyre S. Lived experience of having a child with stroke: a qualitative study. Eur J Paediatr Neurol [Internet]. 2017;21(3):542–8. doi: 10.1016/j.ejpn.2017.01.007 28185801

[21] Plumb P, Seiber E, Dowling MM, Lee J, Bernard TJ, deVeber G, et al. Out-of-pocket costs for childhood stroke: the impact of chronic illness on parents’ pocketbooks. Pediatr Neurol. 2015;52(1):73-6.e2. doi: 10.1016/j.pediatrneurol.2014.09.010 25447931 PMC4276532

[22] Bartha-Doering L, Gleiss A, Knaus S, Schmook MT, Seidl R. Influence of socioeconomic status on cognitive outcome after childhood arterial ischemic stroke. Dev Med Child Neurol. 2020;(December). 10.1111/dmcn.14779 PMC7986130 33336807

[23] Bartha-Doering L, Novak A, Kollndorfer K, Schuler A-L, Kasprian G, Langs G, et al. Atypical language representation is unfavorable for language abilities following childhood stroke. Eur J Paediatr Neurol. 2019;23(1):102–16. doi: 10.1016/j.ejpn.2018.09.007 30314763 PMC6339521

[24] Gomes A, Rinehart N, Greenham M, Anderson V. A critical review of psychosocial outcomes following childhood stroke (1995-2012). Dev Neuropsychol. 2014;39(1):9–24. 24405181 10.1080/87565641.2013.827197

[25] Steinlin M. A clinical approach to arterial ischemic childhood stroke: increasing knowledge over the last decade. Neuropediatrics. 2012;43(1):1–9. doi: 10.1055/s-0032-1307449 22430154

[26] O’Keeffe F, Liégeois F, Eve M, Ganesan V, King J, Murphy T. Neuropsychological and neurobehavioral outcome following childhood arterial ischemic stroke: attention deficits, emotional dysregulation, and executive dysfunction. Child Neuropsychol. 2014;20(5):557–82. doi: 10.1080/09297049.2013.832740 24028185 PMC4104789

[27] Studer M, Boltshauser E, Capone Mori A, Datta A, Fluss J, Mercati D, et al. Factors affecting cognitive outcome in early pediatric stroke. Neurology. 2014;82(9):784–92. doi: 10.1212/WNL.0000000000000162 24489131

[28] Martin M, Jelić A, Doktor Olsen Tvedebrink T. Children’s opportunities for play in the built environment: a scoping review. Child Geogr. 2023;21(6):1154–70. doi: 10.1080/14733285.2023.2214505

[29] Jellema P, Tutenel P, Moser B, Schoss A, Kevdzija M, Jelić A, et al. The space between procedural and situated ethics: Reflecting on the use of existing materials in design research on children affected by stroke. In: Gray C, Ciliotta Chehade E, Hekkert P, Forlano L, Ciuccarelli P, Lloyd P, editors. DRS2024: Boston. Boston, USA; 2024.

[30] Milligan C, Wiles J. Landscapes of care. Pro Hum Geogr. 2010;34(6):736–54. doi: 10.1177/0309132510364556

[31] Mol A, Moser I, Pols J. Care: putting practice into theory. In: Mol A, Moser I, Pols J, editors. Care in practice: on tinkering in clinics, homes and farms [Internet]. Bielefeld: transcript Verlag; 2015. p. 7–26. doi: 10.1515/transcript.9783839414477.7

[32] Ostroff E. Mining our natural resources: The user as expert. Innovation. 1997;16(1).

[33] Blazek M, Hraňová P. Children’s geographies emerging relationships and diverse motivations and benefits in participatory video with young people. Children’s Geographies. 2016;3285.

[34] Ramioul CB, Tutenel P, Heylighen A. Exploring with children what makes a city [Internet]. Vol. 1. Springer International Publishing; 2020. p 99–106.

[35] Walker A, Colquitt G, Elliott S, Emter M, Li L, Walker A. Using participatory action research to examine barriers and facilitators to physical activity among rural adolescents with cerebral palsy. J Phy Act Heal. 2019;8288. 10.1080/09638288.2019.1611952 31088164

[36] Carter B, Ford K. Researching children’s health experiences: the place for participatory, child-centered, arts-based approaches. Res Nurs Health. 2013;36(1):95–107. doi: 10.1002/nur.21517 23192941

[37] Jardine CG, James A. Youth researching youth: benefits, limitations and ethical considerations within a participatory research process.. Int J Circumpolar Heal. 2012;71. 10.3402/ijch.v71i0.18415 PMC3417519 22584512

[38] Jellema P, Annemans M, Heylighen A. Researching and designing health care environments: a systematized review of creative research methods. Qual Health Res. 2019;29(2):290–300. doi: 10.1177/1049732318792227 30111230

[39] Iwarsson S. The housing enabler: an objective tool for assessing accessibility. Br J Occup Ther. 1999;62(11):491–7. doi: 10.1177/030802269906201104

[40] Vermeersch PW, Heylighen A. Mobilizing disability experience to inform architectural practice: lessons learned from a field study. J Res Pract. 2015;11(2):.

[41] Dierckx de Casterlé B, Gastmans C, Bryon E, Denier Y. QUAGOL: a guide for qualitative data analysis. Int J Nurs Stud. 2012;49(3):360–71. doi: 10.1016/j.ijnurstu.2011.09.012 21996649

[42] Mayring P. Qualitative content analysis: basics and techniques. Landsb Beltz. 2010.

[43] Ravens-Sieberer U, KIDSCREEN Europe Group. The KIDSCREEN questionnaires: quality of life questionnaires for children and adolescents; handbook. Pabst Sci Publ. 2006.

[44] Landfeldt E, Zethraeus N, Lindgren P. Standardized questionnaire for the measurement, valuation, and estimation of costs of informal care based on the opportunity cost and proxy good method. Appl Health Econ Health Policy. 2019;17(1):15–24. doi: 10.1007/s40258-018-0418-2 30105745 PMC6346077

[45] Cummins S, Curtis S, Diez-Roux AV, Macintyre S. Understanding and representing “place” in health research: a relational approach. Soc Sci Med. 2007;65(9):1825–38. doi: 10.1016/j.socscimed.2007.05.036 17706331

[46] Wakkary R, Maestri L. Aspects of everyday design: resourcefulness, adaptation, and emergence. Inte J Human-Computer Interact. 2008;24(5):478–91. doi: 10.1080/10447310802142276

[47] Nielsen L, Hansen KS, Stage J, Billestrup J. A template for design personas. Int J Sociotechnology Knowl Dev. 2015;7(1):45–61. doi: 10.4018/ijskd.2015010104

[48] Tvedebrink T, Jelic A. Getting under the(ir) skin: Applying personas and scenarios with body-environment research for improved understanding of users’ perspective in architectural design.. 2018;(November).

[49] Bødker S. Scenarios in user-centred design—setting the stage for reflection and action. Interact Comput [Internet]. 2000;13(1):61–75. https://www.sciencedirect.com/science/article/pii/S0953543800000242

[50] Linden V Van Der, Dong H, Heylighen A. Populating architectural design: introducing scenario-based design in residential care projects. Architectural Design. 2019;13(1):21–36.

[51] World Medical Association. World medical association declaration of Helsinki: ethical principles for medical research involving human subjects. JAMA. 2013;310(20):2191–4. doi: 10.1001/jama.2013.281053 24141714

[52] European Comission. Ethics in social sciences and humanities [Internet]. 2018. Available from: https://ec.europa.eu/info/sites/default/files/6._h2020_ethicssoc-science-humanities_en.pdf

[53] Tutenel P, Ramaekers S, Heylighen A. Conversations between procedural and situated ethics: learning from video research with children in a cancer care ward. The Design Journal. 2019;22(sup1):641–54. doi: 10.1080/14606925.2019.1595444

[54] Angen MJ. Evaluating interpretive inquiry: reviewing the validity debate and opening the dialogue. Qual Health Res. 2000;10(3):378–95. doi: 10.1177/104973230001000308 10947483

