# Supplementary material for: Building support for children and families affected by stroke (BUILD CARE): Study protocol
Source: PLoS One. 2025 Feb 5;20(2):e0308765. doi: 10.1371/journal.pone.0308765 (PMC11798504; doi:10.1371/journal.pone.0308765)
Supplement: S1 Appendix — (PDF) [file pone.0308765.s001.pdf]

Supporting Information 1

Work Package 2 - Part 1: Demographic questions

1. General Information

Household	Collected Data
Child	Sex<br>Year of Birth<br>Year of Stroke
Person completing the questionnaire	Sex<br>Year of Birth<br>Highest level of education:<br>1. No compulsory school certificate<br>2. Secondary level I: Completion of a Hauptschule, Realschule, Gesamtschule, Mittelschule, Pflichtschule, AHS- Unterstufe<br>3. Secondary level II: Completion of a grammar school, specialised secondary school, vocational (technical) school, AHS upper secondary school, polytechnic school, BHS, BMS<br>4. Post-secondary and tertiary level: Degree from a university or university of applied sciences (bachelor's, diploma, master's, state examination, doctorate) or master craftsman training or equivalent educational programme<br>5. Other qualification<br>Working hours per week

	Occupation (according to ISCO- 8):<br>1. Armed Forces Occupations<br>2. Managers<br>3. Professionals<br>4. Technicians and Associate Professionals<br>5. Clerical Support Workers<br>6. Service and Sales Workers<br>7. Skilled Agricultural, Forestry and Fishery Workers<br>8. Craft and Related Trades Workers<br>9. Plant and Machine Operators, and Assemblers<br>10. Elementary Occupations
Other parent	Sex<br>Year of Birth<br>Highest level of education (see above)<br>Occupation (according to ISCO- 8) (see above)
Other children	Y/N<br>Age group:<br>□ Newborn (0 - 28 days)<br>□ Infant (29 days to 12 months)<br>□ Toddler (13 months to 3 years)<br>□ Child (4 years to 12 years)<br>□ Teenager (13-18 years)
Other household members	Y/N<br>Number of persons of legal age

2. What symptoms and limitations are noticeable in your child's everyday life?

- ◯ Hemiparesis:
  - ◯ of the arm and/or
  - ◯ of the leg
- ◯ Seizures
- ◯ Impaired consciousness
- ◯ Impaired speech
- ◯ Impaired sensory perception
  - ◯ Taste
  - ◯ Smell
  - ◯ Vision
  - ◯ Hearing
  - ◯ Balance
  - ◯ Touch
  - ◯ Pain perception
  - ◯ Temperature perception
  - ◯ Other: ___________
- ◯ Behavioural changes
- ◯ Facial palsy
- ◯ Walking impairment
- ◯ Orientation difficulties
- ◯ Other symptoms: ______________________________

3. Which side of the brain is affected?

◯ left hemisphere ◯ right hemisphere ◯ both sides
