# Supplementary material for: Building support for children and families affected by stroke (BUILD CARE): Study protocol
Source: PLoS One. 2025 Feb 5;20(2):e0308765. doi: 10.1371/journal.pone.0308765 (PMC11798504; doi:10.1371/journal.pone.0308765)
Supplement: S2 Appendix — (PDF) [file pone.0308765.s002.pdf]

Supporting Information 2

Work Package 2 – Part 4: Questions related to the built environment

1. How long have you been living in your home?

2. Did you have to move because of your child's needs?

◯ yes ◯ no

2.1. Do you rent or own the home?

◯ rent ◯ own

3. Did you have to make any modifications to your current home?

◯ yes ◯ no

3.1. If yes, where?

Multiple Choice, multiple answers possible:

◯ entrance area ◯ kitchen ◯ living area
◯ bathroom ◯ children's room ◯ no modification
◯ others:____

3.2. What was the reason for the modification?

Multiple Choice, multiple answers possible:

◯ needs of the child ◯ barriers in everyday life ◯ accessibility
◯ usable with one hand ◯ more independence for the child ◯ recommendations from therapist, other families, etc.
◯ other reasons:____

3.3. Has the modification proven helpful?

◯ yes ◯ no ◯ I don't know

3.4. Is there a modification to your home you would like to do but have not yet done?

Open-ended

3.5. If yes, what is the reason for not implementing this modification?

Multiple Choice, multiple answers possible:

- ◯ financial reasons
- ◯ time-related reasons
- ◯ structure of the building
- ◯ renting or other legal reasons
- ◯ no design ideas for improvement
- ◯ other reasons:____
